# Community‐Driven Health Promotion: Evaluation of a Rural Microgrant Program

**DOI:** 10.1111/hex.70098

**Published:** 2024-11-10

**Authors:** Michele Conlin, Dorothy McLaren, Evelien Spelten, Sean MacDermott

**Affiliations:** ^1^ La Trobe University Rural Health School Bendigo Victoria Australia; ^2^ West Wimmera Health Service Nhill Victoria Australia; ^3^ John Richards Centre for Rural Ageing Research Bendigo Victoria Australia

**Keywords:** community participation, empowerment, microgrants, program evaluation

## Abstract

**Introduction:**

Microgrants for health promotion have the potential to engage communities in designing and implementing place‐based interventions to improve health and social outcomes. However, the evidence base around microgrants for health promotion is limited. This study presents the evaluation of a health service‐funded microgrant program for health promotion in rural Australia.

**Methods:**

The evaluation framework was developed through a participatory approach and involved collaborative logic model building and co‐prioritized evaluation questions with health service staff and grant recipients. Evaluation questions focused on participation, short‐term outcomes, and the perceived value of program activities. Qualitative methods (reflexive thematic analysis) were used to answer the evaluation questions. Data sources included semi‐structured interviews with grantees (*n* = 11) and the health service's health promotion team (*n* = 4), electronic field notes kept by the health promotion team (*n* = 50 documents), electronic progress reports completed by grantees (*n* = 6) and information and feedback forums (*n* = 2).

**Results:**

Since the program's inception in 2019, the health service has received 22 grant applications of which 15 were approved and 14 disbursed. Evaluation results show that grantees were community members with multiple roles, often with previous experience in applying for grants. Expected outcomes have been partially met, especially with regard to the program's aim of community empowerment. The most notable impact was the enhancement of participants' perception of and relationship with the health promotion team, as well as the creation of opportunities for community members such as exposure to art and bridging of social groups.

**Conclusion:**

Microgrants represent a feasible way to increase health opportunities and foster community participation in the planning and delivery of health promotion programs. The key program activities identified and suggested improvements can help guide program replication and adaptation by other small organizations.

**Public Contribution:**

Community members who had previously received a health service grant were invited to participate in collaborative workshops and follow‐up surveys to codesign the grant program evaluation framework, co‐prioritize evaluation questions and guide the results' dissemination plan.

## 1 Introduction

Microgrants have become an increasingly common approach for driving innovative, community‐led initiatives across sectors. They are defined as small cash awards given to community groups to enable them to design and implement locally relevant projects [1]. Health promotion (HP) practitioners are one of the many groups to adopt microgrants as a strategy to stimulate place‐based interventions, with aims ranging from increasing physical activity levels and addressing chronic disease risk factors, to enhancing youth involvement in community activities and fostering collaborative partnerships, among many other objectives [2]. The processes involved in microgrants for HP resonate with some of the core principles of the discipline, notably enabling, supporting, and empowering individuals and communities to improve their health and social outcomes [3]. This is exhibited in the microgrants' shifting of deciding power about what interventions should be implemented back to the communities themselves, leading, in theory, to more acceptable and effective HP activities. Unfortunately, robust evidence demonstrating the HP potential of microgrants is currently limited, particularly for rural settings [2].

Evaluation of public health and health promotion interventions can be challenging. Practitioners are frequently faced with multiple barriers, such as a lack of knowledge and skills in evaluation methods, and time and funding constraints [4, 5]. Consequently, the quality of program evaluations may suffer, especially when undertaken by smaller organizations, as highlighted by Schwarzman et al., in their assessment of health prevention agency evaluations [6]. It is therefore of no surprise to find few high‐quality evaluations of microgrant programs for HP [6], as the usual evaluation challenges are compounded by the multi‐level domains in which these programs operate (i.e., the funding agency, community contexts, grant recipients and collaborators, and funded activities and their participants). Nonetheless, these programs may have HP potential, with the few available studies reporting positive impacts on intersectoral collaboration, local leadership, community initiatives and changes, access to community resources and engagement with non‐health‐related organizations [7, 8, 9, 10, 11, 12].

Another weakness (or rather, missed opportunity) in the available research on microgrants for HP is the infrequent engagement of community stakeholders in program evaluations. In this team's previously published scoping review [2], only two of 18 microgrant evaluations were reported involving different stakeholder groups as part of participatory evaluation processes [9, 11]. Not only are participatory evaluation approaches in line with the foundations of HP practice but they also represent an HP strategy in themselves [13]. Additionally, they may contribute to capacity building within organizations and the broader community, as well as improve the meaningfulness and utility of findings [5, 14, 16].

**Figure 1:**
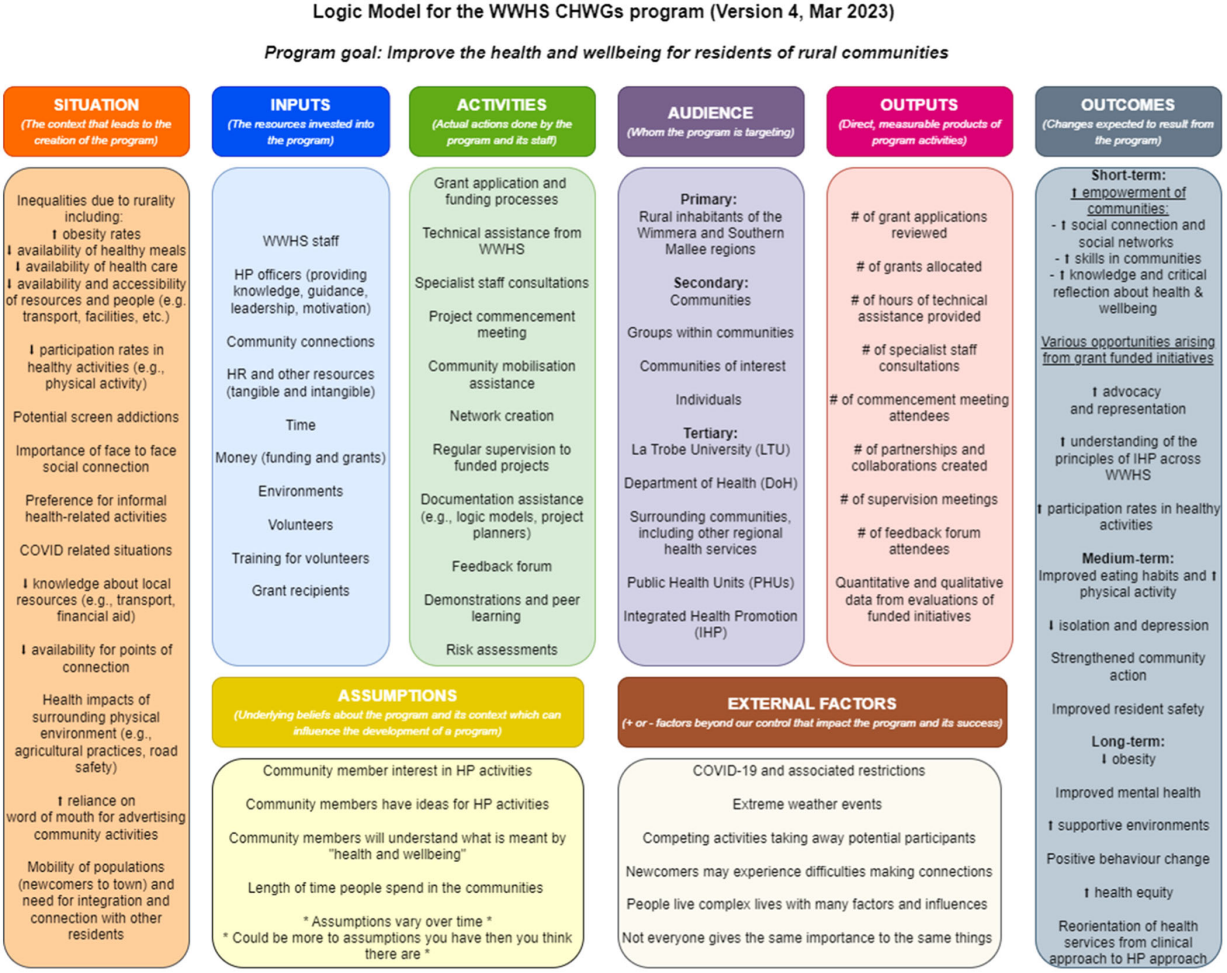
The microgrant program logic model was developed collaboratively with grant recipients, health service staff and the research team.

With the aims of addressing both the limited evidence base around microgrant programs for HP in rural settings and the missed participatory opportunities from previous evaluations, this study evaluates a rural, health service‐funded, microgrant program using a participatory approach (codesign [16]) and guidance from public health and health promotion evaluation frameworks [17, 18, 19, 20]. In keeping with the focus of codesign, which draws on a variety of stakeholders' insights to establish priorities and plans [16], health service staff and grant recipients were involved in building the program's logic model (Figure 1) and defining the evaluation's goals. As such, the primary objective of this study is to answer the three evaluation questions selected during the codesign processes:

Who participates in the microgrant program?

What are some short‐term outcomes of the microgrant program?

What value do participants attribute to the various program activities?

Secondary aims of this study are to provide recommendations for program replication and adaptation in small organizations looking for new ways to engage with local communities in health promotion efforts.

## 2 Materials & Methods

### 2.1 Intervention

The microgrant program is funded and coordinated by a local health service in rural Australia. The health service serves a mainly agricultural area, which includes nine communities with diverse health profiles, with a total population of approximately 16,000 (Modified Monash category 5, or “small rural towns” [21]). The health service's health promotion (HP) team (4 full‐time equivalents) dedicates approximately a 0.6 full‐time equivalent to the microgrants. Allied health professionals from the health service (e.g., dieticians, nurses, physiotherapists, podiatrists) occasionally support grant‐funded activities when relevant (e.g., presentations on healthy eating, foot care, blood pressure checks, etc.). The first grant ground was launched in 2019; however, the 2021 grant round was cancelled due to concerns about COVID‐19 risks and restrictions, and the 2023 grant round was postponed to September of that year.

The grants (up to AU$5000) are available to individuals or community groups and can fund once‐off projects (e.g., a health fair) or recurring activities (e.g., a weekly activity). Applications consist of a short online form, with assistance provided by the HP team manager to refine ideas and complete the form. Applications are then reviewed by a deciding committee made up of an HP officer (excluding the team manager), an academic partner and a senior health service executive, and utilize an internally developed scoring sheet. Each successful applicant is assigned an HP officer and regular meetings are established to ensure the successful implementation of the funded project. In terms of reporting, each grant recipient is asked to complete periodic progress reports; however, there is no mandatory reporting as a condition to receiving funds.

### 2.2 Evaluation Framework

The evaluation framework was developed through codesign processes with health service staff (HP team and one executive) and grant recipients. Activities included semi‐structured interviews, focus groups, logic model (LM) building workshops and surveys. Full details of the framework's codesign are described elsewhere [22]. As stakeholders voiced a preference for a theory‐informed evaluation, the program LM became the basis for the framework (Figure 1), and the prioritized evaluation questions correspond to different components of the model (*audience* [Q1], *outcomes* [Q2], and *situation, inputs*, and *activities* [Q3]). The second evaluation question (“*What are some short‐term outcomes of the microgrant program?”*) prompted discussions around the importance of looking at both expected outcomes (as per the LM) and unexpected outcomes (not appearing in the LM). Additionally, the expected outcomes outlined in the LM required clarification and operationalization, particularly the outcome of *community empowerment*, which was done during a workshop with the HP team (appearing in Figure 1) [22]. *Community empowerment* and the *creation of opportunities for health through funded activities* were chosen by the HP team as the expected short‐term outcomes to focus on for the current evaluation. Table 1 provides an overview of indicators, data sources, methods and timeframes for the evaluation.

**Table 1:** The microgrant program evaluation framework.

**Logic model component**	**Co‐prioritized evaluation question**	**Indicators**	**Data sources, methods**	**Study population**	**Time point**
Audience	Q1: Who participates in the microgrants?	*Quantitative:* # of grant applications received, # of grants allocated/refused, # of information and feedback forum attendees.	HP team documentation (grant applications), observation, individual interviews	Information and feedback forum attendees, grantees	Yearly (forums) & throughout 2023
		*Qualitative:* Applicant time in community/region, motivations, experience, feedback forum queries.			
Outcomes	Q2: What are the short‐term outcomes of the CHWGs?	*1a. Community empowerment − community self‐capacity and self‐determination:* Perceived increase in capability and an increased sense of control over processes and decisions that affect community health (grantees), increase in participation in community projects before/after grant program involvement (grantees), interest in future participation (grantees).	HP team documentation (field notes), observation, individual interviews, grantee progress reports	Information and feedback forum attendees, grantees, HP team	Yearly (forum) & throughout 2023
		*1b. Community empowerment − positive relationships and multisectoral collaborations:* Increased awareness of community groups working in “health” (grantees), new previously unexplored relationships between groups during or after funded activity implementation (grantees), reports of positive outcomes from new relationships (grantees), improved perceptions of WWHS as a resource for health (grantees) and increased likeliness of community members to utilize health services.			
		*1c. Community empowerment − community knowledge and capacity for critical reflection on community health:* Increased knowledge of the social determinants of health (grantees and community members), increase in conversations about health and wellbeing and/or the social determinants of health (grantees and community members).			
		*2. Opportunities for health arising from grant‐funded initiatives as reported by grantees and HP staff:* Outcomes of funded projects as compared to initial expectations, reported highlights and lowlights and reported surprises at any stage (planning, implementation, post‐implementation, etc.).			
Situation, Inputs, Activities	Q3: What value do participants attribute to the various program activities?	Value of program activities as perceived by participants.	Individual interviews	Grantees, HP team	Throughout 2023
		Perceived usefulness/appropriateness of program in tackling local health issues.			
		Perceived transferability of program.			

### 2.3 Data Collection

To answer the evaluation questions, data was derived from four sources:

1. *Semi‐structured interviews*: Eleven grantees were interviewed resulting in an 85% (11/13) response rate. Interviews were one‐on‐one except for one instance where two individuals from the same organization attended. One grantee who was involved in two projects was interviewed about both in a single interview. All four HP personnel were interviewed. Interviews were conducted between May and August 2023, lasted from 21 to 71 min and occurred in‐person, by phone or videoconference. Interviews were recorded with participants' consent, transcribed verbatim and deidentified. Interview guides are available in the Online Supporting Materials S1.
2. *Electronic field notes*: The HP team's electronic field notes (*n* = 50 files) documented phone and in‐person meetings with grantees ranging from November 2019 to March 2023. Five different HP team members contributed to the field notes across this time period, three of which were still employed at the time of writing (and also interviewed). Field notes were not completed in a consistent fashion across all team members.
3. *Electronic progress reports*: Formal grantee progress reports were collected at a single time point by the health service (April–May 2023). These were completed electronically by six grantees from all three grant rounds.
4. *Observation during online forums*: Two online community microgrant information and feedback forums were organized by the health service. The first was held in 2022 and was attended by six community members. The second was scheduled in 2023 but was not attended by any community members.

Data collection, including interviews, was conducted by one researcher (MC).

### 2.4 Data Analysis

NVivo 14 software [23] was used to facilitate the analysis. Interview transcripts, field notes and progress notes were reviewed independently by three researchers (MC, SM, ES). Overarching themes were then identified through consensus discussions. Further reflexive thematic analysis [24, 25] was applied by one researcher (MC), firstly using deductive coding with LM components as primary nodes (e.g., *situation*, *inputs*, *activities*, etc. [Figure 1]). Themes were then identified inductively within each of these nodes. For program outcomes, the inductively identified themes were then categorized as expected or unexpected according to what was detailed in the LM. Final coding was reviewed independently by SM and ES and alternate interpretations were discussed and resolved through consensus. As a last step, member‐checking was used to increase the trustworthiness of the findings.

### 2.5 Ethics

All research activities were reviewed and approved by the La Trobe University ethics committee (HEC20505). Informed consent was obtained through REDCap electronic data capture tools hosted at La Trobe University [26], as well as verbally from all participants before interviews.

## 3 Results

### 3.1 Evaluation Q1: Who Participates in the Microgrant Program?

Twenty‐two grant applications were received across the 2019, 2020 and 2022 grant rounds, of which 15 were approved. One was eventually withdrawn due to the applicants finding funding elsewhere. Most were awarded to community organizations for ongoing or sustained activities (Table 2). Reasons for not awarding a grant included projects that were outside the scope of health promotion, needed ongoing financial support or for which the main use of funding was infrastructure.

**Table 2:** Grant‐funded project descriptions and reported outcomes.

**Grant round**	**Grant recipients**	**Project type**	**Grant amount**	**Project description**	**Outcomes of funded activities as reported by grant recipients** a
2019	Individual community member(s)	One‐off event	$4890	Community consultation to assess interest in the development of walking and biking tracks	Attended by 48 community members, including young families and older adults; report produced from the consultation was leveraged to obtain a larger grant for project implementation.
2019	Community organization	Ongoing	$5000	Community garden	Project was on hold at the time of writing due to unexpected personal circumstances.
2019	Community organization	Ran for two years	$5000	Various activities aiming to increase the use of local public pools (invitations to closed pool days, aqua training, activities for schools, workshop with dietician, free pool access for low‐income families)	Intended to run for 5 years but ended after 2 due to change in management; difficulty in attracting new users; aqua training and dietician workshop well attended (numbers not recorded).
2019	Individual community member(s)	Ongoing	$5000	Community garden	Garden maintained by a group of approx. 10–12 local women; local primary school and nursing home have organized visits to the garden; a free Open Garden Day BBQ attracted approx. 100 residents; plants and equipment are regularly donated by community members.
2019	Individual community member(s)	One‐off	$3000	Low‐cost 11‐week exercise and health & wellbeing program aimed at firefighters and farmers (but open to all community members)	Disrupted by the start of the COVID‐19 pandemic, the last 4 weeks were modified to an online format; 32 people enroled; pre‐post health data collection planned, but post data not obtained due to COVID‐19 restrictions; program coordinator received ample positive feedback during follow‐up phone calls.
2019	Local government	One‐off	$5000	Free community concert and health and wellbeing expo to connect people, services and opportunities (included a variety of interactive presentations and activities)	Event was required to split into the morning (youth only) and afternoon (all‐ages) sessions due to legal barriers; attended by approx. 100 14–17‐year‐olds in the AM and 50 mainly older adults in the PM; Notable absence of middle‐aged adults (30‐50 age range); Presenters from 8 regional community organizations and health services.
2020	Individual community member(s)	Ongoing	$5000	Various activities to support the use of existing local physical activity assets and foster social connection	Disrupted by COVID‐19 restrictions; project being redefined between grant recipients and the HP team at the time of the interview.
2020	Community organization	Ongoing	$3000	Weekly Qigong classes (requiring gold coin donation) and periodic “paint and sip” workshops (reduced fee)	50+ Qigong classes delivered so far with approx. 8 people/class rotating out of 20 semi‐regular attendees (mainly retired females); Qigong classes have also become places of social connection and garden produce exchange; 5 “paint and sip” workshops held so far with approx. 30 participants total, and more workshops are planned targeting different age groups.
2020	Community organization	Ongoing	$5000	Health and wellbeing website and committee creation for the local sporting club (with public access to the website)	Health‐related articles were posted periodically on the website; difficulty in recruiting committee members; workshop was held by a dietician with junior club members about healthy eating; traffic light system was implemented in the club canteen (with help from the dietician).
2020	Individual community member(s)	Ongoing	$5000	Mosaic art workshops with pieces becoming features around town and forming a discovery trail	Workshops run weekly on most weeks (with some interruptions due to personal circumstances); 8 regular workshop attendees and 2 semi‐regular (all female, intergenerational); 18 mosaics featured around town so far.
2022	Community organization	Ongoing	$5000	Promotion of a local walking track through the installation of a photo trail of regional silo art, including interpretation of silo history and local crop production	Project in the implementation phase at the time of the interview.
2022	Community organizations	Ongoing	$5000	Community garden	Project in the implementation phase at the time of the interview.
2022	Community organization	Ongoing	$1650	Yearly welcome dinners for newcomers to town	Two dinners held so far, first very well attended (approx. 100 people) and second less so (exact numbers not recorded); events were intergenerational with multiple community groups intermingling.
2022	Community organization	One‐off	$1500	Two expert‐led art workshops at a reduced price	Workshops planned but not yet held at the time of interview; both workshops filled immediately after publicizing (8 people/workshop) with intergenerational attendance (from 10 years old +).

a Due to the ongoing nature of some of the funded activities, outcomes may have changed since the time of the interview.

Applications were received from community members and organizations from eight of the nine communities in the health service catchment. The main applicants were all female and those interviewed identified themselves as long‐standing members of their communities. Eight of the 12 interviewed grantees stated having experience in applying for grants in the past, whereas levels of experience in organizing community projects or activities varied. Three of the participants were currently or previously employed by the health service. Most grantees mentioned having multiple roles in addition to being involved with their funded projects, for example, being employed full‐time, volunteering, having young children in their care and/or caring for elderly parents. When discussing their motivations behind applying for health service grants, several grantees identified their desire to support mental wellbeing and social connections in their communities.

### 3.2 Evaluation Q2: What Are the Short‐Term Outcomes of the CHWGs?

#### 3.2.1 Expected Outcome #1: Community Empowerment

a. Community self‐capacity and self‐determination: This domain was assessed at the level of the individual grantees and their immediate collaborators (e.g., individuals who helped implement funded activities). In this regard, outcomes were mostly positive or neutral (no reported gains). Half of the participants expressed having more confidence in applying for grants and/or running community projects or having learned new skills throughout these processes. Only one participant stated they were less likely to apply for grants in the future, mainly related to a sense of burden at having taken on the coordinating role for the project. Of note, two grant recipients identified already having extensive experience with grant writing and coordinating community projects.
  ‘And it [the grant] gives us confidence too, because you know, we're not… We've all had other jobs. We're cleaners and teachers and nurses and… […]. So it will do us good to think, oh we could actually do something like this too.’ – Grantee
b. Positive relationships and multisectoral collaborations: When discussing community connections that may have resulted from planning and implementing funded projects, participant responses varied between positive and neutral. About half of the grantees reported having developed meaningful relationships with new individuals or groups during their experience.
  ‘Interviewer: But were there any new connections that were formed because of this project?
  Grantee: Um, yes. The [Smithtown] Neighborhood House. […] And I didn't know they existed until then. And we've done lots of projects with them since for youth and students.’
  Eight of the grantees, however, commented on how their awareness of services available through the health service increased and their relationship with the HP team was enhanced. Most mentioned they felt more comfortable approaching the HP team after their experience or were more likely to turn to the team for support or collaborations with future projects.
  ‘I probably, yeah, got more of an idea of what services we can get in town now, um, through [the HP team manager] and… what yeah, what are the benefits we can, we can have for the community through the [health service].’ – Grantee
  Two participants who were long‐standing members of their communities did not report any new connections with either individuals, community groups, or the health service.
c. Knowledge and capacity for critical reflection on community health: This outcome was the least successful, with mostly neutral responses from participants. Reflections tended to be at the individual level with a few broader openings about the positive impacts of social connections and the social determinants of health. Some participants directly stated that they had not seen much impact at this level.

‘We're having a little bit [of conversations about community health], but not big, like it hasn't been a big thing to focus on health. The main thing […] was to get people out, outside. So I suppose that is a little way community health, but not that big broader…’ – Grantee

#### 3.2.2 Expected Outcome #2: Opportunities for Health

A recurring theme across both grantee and staff interviews was how the microgrants were facilitating the creation of opportunities for a variety of people in small communities.

‘Like it's [the grant program] literally asking the people what they want. So… And I think particularly people in rural areas, they don't always get what they want as such. They're sort of deprived of a lot of things.’ – HP officer

A few grantees mentioned how their projects were helping to address the lack of opportunities for local youths specifically, and other interviewees felt that some of the projects tackled what was seen as the cliquish nature of some small country towns.

‘And also in the first [funded art workshop], there's four children […] who are really interested in art, but there's no groups that they can go to, they're too far away from [the regional hub], really.’ – Grantee

‘Like there's the pub, there's the church, there's the footy, and netball and there's the [other sport group] and… you know. But now it seems like some of the local people turned up [to the Welcome dinner], especially the ones that were involved, and you know made connections […], and that was the point.’ – Grantee

### 3.3 Unexpected Outcomes

Few unexpected outcomes were identified. One grantee reported having been surprised by negative interactions and behaviours during the course of their project, notably having trolls in the online community group and having one of their artworks vandalized. A more positive unexpected outcome was how a home produce exchange developed within a series of funded exercise classes.

### 3.4 Evaluation Q3: What Value Do Participants Attribute to the Various Program Activities?

Other than the grant money itself (which was frequently elicited as having an obvious value), a recurring theme was how the health service grants stood apart from other small grants available to rural communities in terms of the support available to applicants. Grantees almost unanimously reported having received help from the HP team to shape their initial ideas and complete the grant application. This was often perceived as a pivotal part of the grant process, contributing to dissolve applicants' doubts about their ability to complete the application and to qualify for the funding. Furthermore, most grantees expressed feeling supported throughout the planning and implementation phases of their funded projects, with HP officers being available by phone or in‐person (outside of COVID restrictions). The HP team often provided technical assistance in terms of advertising needs (e.g., printing flyers) or providing resources (e.g., directing towards potential sources of additional funding). Overall, the hands‐on approach, combined with team availability and enthusiasm, was highly valued by the interviewed grant recipients.

‘I felt like the process was very easy…compared to some of the grants that I've put in […]. Because a lot of times you spend a lot of time putting in grant applications and not getting anything back.’ – Grantee

‘Because they're [the HP team] very open to remaining involved and supportive of where it's gone to next. […] That's probably been a highlight of this actual grant process.’ – Grantee

## 4 Recommendations for Program Replication and Adaptation

Program strengths and weaknesses were identified throughout the analysis. These have been summarized in Box 1 and provide key considerations for program replication or adaptation. Additionally, specific evidence‐based strategies have been included to further support the intended program outcome of enhancing grantee and participant knowledge and capacity for critical reflection on community health [27, 28, 29].

Box 1 **Considerations for Program Replication and Adaptation**

*Key program activities provided by the health promotion (HP) team:*

- Strong guidance by the HP team during initial contacts with community members considering applying for a grant.
- Initial assistance in refining applicants' ideas as well as hands‐on help with drafting grant applications.
- Tailored support and facilitation for funded project implementation, building on grant recipients' skills and resources.
- Providing information about local resources including those available from the health service.
- Ensuring continued alignment of funded activities with principles and values of health promotion.
- Supporting project sustainability when appropriate, including assistance with finding additional sources of funding.
- Assistance with advertising of funded activities.
- Ongoing contact with grant recipients (including post‐implementation of funded projects) to maintain newly established community connections.

*Elements identified for program improvement:*

- Clear procedures, responsibilities and timelines for staff and grantees.
- Structured debriefing between the HP team and grantees once projects have been implemented.

*Suggested activities to foster knowledge and critical thinking on community health:*

- Formal training for HP staff on enhancing their own critical thinking and on developing teaching strategies to be applied during meetings with grant recipients [27, 28].
- Increased focus on the influence of policy and environmental determinants of health in the grant application process and during initial discussions with grantees when planning funded activity implementation [27, 28].
- Integrating short, innovative activities (e.g., role‐playing games) with funded activity participants to enhance critical thinking skills and health literacy surrounding community health issues [29].

## 5 Discussion

The health service‐funded microgrant program evaluated in this study represents a bottom‐up approach to engage with small, rural communities and codesign health promotion activities. A participatory evaluation was built to further the opportunities for connections and empowering processes [22]. Four years post‐implementation, 14 microgrants have been disbursed from 22 applications. Evaluation results show that grant recipients were active community members with multiple roles, often with previous experience applying for grants. Expected outcomes have been partially met, especially with regard to the program's aim of community empowerment. The most notable impact was on participants’ perception of, and relationship with, the health service's HP team, and the creation of opportunities for community members that were thought to be otherwise lacking (e.g., exposure to art through painting or mosaic workshops, or bridging of social groups through Welcome dinners). The weakest impact was on participant's knowledge and capacity for critical reflection on community health and the social determinants of health. Finally, the summary of the program's key activities and areas for improvement can provide a blueprint for practitioners looking to replicate or adapt the microgrant program to stimulate community‐driven HP activities.

Comparison of evaluation results across different HP microgrant programs is challenging due to the broad range of activities, indicators and measures [2, 12]. For example, Tompkins et al.'s evaluation focused on the types of grant recipients (health vs non‐health related groups) and funded project focus areas (policy, environment, or system) [12]. In contrast, Baker et al. assessed both individual and community‐level outcomes, of which the creation of diverse opportunities for physical activity (the microgrant target), alongside increased participation in local events and increased life skills were evaluation targets [11]. Ramanathan et al. similarly assessed a wide range of outcomes, reporting benefits in personal development, organizational capacity and relationship building, as well as increased opportunities for physical activity (the microgrant target) [30]. Despite the heterogeneity of evaluations, the findings of this study align with those of Baker et al. and Ramanathan et al., in terms of microgrants' potential to build skills and capacity in individuals and organizations, as well as to create opportunities and increase access to resources for health [11, 30].

Another complexity in microgrant evaluation is the often limited resources of grant recipients to evaluate their funded projects, translating to sparse evidence around the impact of these projects [31, 32, 33]. Gabbert et al. argue that expecting high‐level reporting and evaluation from small community organizations is generally inappropriate [33]. This was a sentiment shared by the health service team during initial evaluation planning, and thus no formal evaluation measures were required from grant recipients. However, with the current evaluation results having sparked conversations with the HP team and grant recipients about growing capacity for evaluation, future data collection activities are planned to incorporate a closer look at the impacts of individual projects, whilst still aiming to not over‐burden grant recipients with evaluation tasks.

### 5.1 Strengths and Limitations

The strength of this evaluation lies in its use of a co‐designed framework derived from the program LM and evaluation questions, which were prioritized between the academic research team, the health service's HP team and several grant recipients. Furthermore, findings were made available to participants to review and comment on before dissemination. The results of the evaluation will provide constructive and actionable feedback to the health service, as well as promote discussion about potential improvements to help achieve currently unmet outcomes. Additionally, the dissemination plan for this evaluation includes health service and community‐level outlets to encourage conversations around the HP team's work.

As for limitations, it is important to note that only three grant rounds have been held at this time, all in the context of a global pandemic. Continued monitoring and evaluation are needed to assess the impact of the program over time, especially focusing on broader, community‐level impacts. Although a longitudinal design was beyond the scope and funding of this study, all evaluation materials and resources used throughout the study, as well as additional tools to support the evaluation of individual funded activities, have been made available to the HP team to support continued evaluation efforts. Finally, the use of the microgrant program‐specific LM for evaluating outcomes may have simplified an otherwise complex landscape in which the HP team's broad portfolio of programs intersects and interacts. Further workshops are planned with the health service to explore these interactions in more depth.

## 6 Conclusion

This study grows the evidence base for microgrants for rural health promotion, having applied a co‐designed evaluation framework to answer stakeholder‐prioritized evaluation questions. Although expected program outcomes were only partially achieved, the evaluation highlighted how the microgrants facilitated new and/or enhanced relationships between grant recipients and the health service, potentially leading to further collaborations in the future. As such, microgrants represent a feasible way to increase opportunities for health in rural settings, as well as foster community participation in the planning and delivery of health promotion programs. The key program activities identified and suggested improvements can help guide program replication and adaptation by other rural organizations looking to implement a similar program.

## Author Contributions

**Michele Conlin:** conceptualization, investigation, writing–original draft, methodology, validation, visualization, writing–review and editing, formal analysis, project administration, data curation. **Dorothy McLaren:** conceptualization, methodology, validation, writing–review and editing, project administration, supervision. **Evelien Spelten:** conceptualization, methodology, validation, formal analysis, supervision. **Sean MacDermott:** conceptualization, methodology, validation, formal analysis, supervision.

## Ethics Statement

All research activities were reviewed and approved by the La Trobe University ethics committee (HEC20505). Informed consent was obtained through REDCap electronic data capture tools hosted at La Trobe University, as well as verbally from all participants before interviews.

## Conflicts of Interest

The authors declare no conflicts of interest. However, please note that this study used a codesign approach, and as a result, Dorothy McLaren had two roles: participant and author.

## Supporting information

Supporting information.

## Data Availability

The data that support the findings of this study are available from the corresponding author upon reasonable request.
