# Supplementary material for: Community‐Driven Health Promotion: Evaluation of a Rural Microgrant Program
Source: Health Expect. 2024 Nov 10;27(6):e70098. doi: 10.1111/hex.70098 (PMC11551473; doi:10.1111/hex.70098)
Supplement: Supplementary file 1 — Supporting information. [file HEX-27-e70098-s001.docx]

**Supplemental materials 1**

INTERVIEW GUIDE FOR SEMI-STRUCTURED INTERVIEWS WITH MICROGRANT RECIPIENTS

“I’ve prepared a few questions to better understand your experience in participating in West Wimmera Health Services’s Community Health and Wellbeing Grants program. Your answers will be kept anonymous. I would however like to record our conversation so I can transcribe it and re-read it when I prepare my report. I will ensure all names are removed from the transcript and will send it to you for your review. Do I have your consent to do so?”

*Intro Qs*

1. How long have you lived in your current community? And in the region?
2. Tell me about your community project…

*Eval Question 1: Participation*

1. What motivated you to apply for a grant?
2. Have you had any previous experience in applying for community grants? In running community projects? (If so, please elaborate).

*Eval Question 2: Short-term outcomes*

1. When you applied for the grant, you had to define what success for your project would look like. What were your initial expectations, and were these expectations met?
2. Did anything surprise you during the planning and execution of your project? (What was it? Why was it surprising to you?) Did you have any unexpected conversations that have stayed with you (i.e. that did not directly relate to your project)? If so, about what?
3. Who were you hoping to engage or attract with your project/activity(ies)? Who actually turned up? (Were they familiar faces, new faces, both?)
4. What were the highlights and low points during your time involved with the grant program?

*Community self-capacity and self-determination*

1. At the beginning of a new activity, we often don’t know what we don’t know and it’s only by the time that we reach the end that we feel that we could now tackle the task properly. Given that, what advice would you offer to someone else undertaking a similar project now?
2. Has this experience affected whether or not you would apply for another grant? Are you more or less likely – why? (What would need to happen to shift you from less likely to more likely?)

*Positive relationships and multisectoral collaborations*

1. Have you made any new connections in your community during or after your participation in the grant program? (If so, with whom? How did this connection happen?)
2. Has your opinion of West Wimmera Health Service changed in any way? (If so, how?)

*Community knowledge and capacity for critical reflection on community health*

“West Wimmera Health Service’s Community Health and Wellbeing Grants' overall goal are to improve individual and community health…”

1. Have you learned anything new about community health through your participation in the grant program? (If so, please elaborate…)
2. Roughly how many conversations about community health have you had with other community members in the past 6 months? Would you say this is more/less/about the same as “usual”? (If there’s been a change, what do you think is the reason?)

*Eval Question 3: Program activities*

1. What were the most and least useful aspects of the grant program in helping you plan and implement your project?
2. Was the program support a barrier or facilitator in achieving your desired outcomes?
3. In general, do you think the grant program helps tackle local health issues? (If so, which problems and in what way does it help?)
4. Do you think this type of grant program could be helpful in other rural communities across the country? Why or why not?

*Closing Q*

1. Is there anything that we haven’t discussed so far that you would like to talk about?

INTERVIEW GUIDE FOR SEMI-STRUCTURED INTERVIEWS WITH HEALTH PROMOTION OFFICERS

“I’ve prepared a few questions to better understand your role and experience with your team’s Community Health and Wellbeing Grants (CHWG) program. Your answers will be kept anonymous. I would however like to record our conversation so I can transcribe it and re-read it when I prepare my report. I will ensure all names are removed from the transcript and will send it to you for your review. Do I have your consent to do so?”

*Intro Qs*

1. How long have you been involved with the CHWGs?
2. Tell me about your role in the program…

*Eval Question 1: Participation*

1. Who is being targeted by the CHWG program?
2. In your experience, who has been applying for the CHWGs? Is this your target audience (as described above)? If not, how so?

*Eval Question 2: Short-term outcomes*

1. Throughout your work with grantees, what are some of the impacts the CHWGs have had on local communities that you’ve observed? Please provide examples.
2. In your opinion, is the CHWG program meeting its expected outcomes? (*Referring to the logic model*). Why or why not?
3. Has anything surprised you during your experience with the CHWGs? (What was it? Why was it surprising to you?)

*Eval Question 3: Program activities*

1. In your opinion, what are the strengths and weaknesses of the CHWG program?
2. What improvements could be made to the program?
3. In general, do you think the grant program helps tackle local health issues? (If so, which problems and in what way does it help?)
4. Do you think this type of grant program could be helpful in other rural communities across the country? Why or why not?

*Closing Q*

1. Is there anything that we haven’t discussed so far that you would like to talk about?
